# Protein and Amino Acid Content in Four Brands of Commercial Table Eggs in Retail Markets in Relation to Human Requirements

**DOI:** 10.3390/ani10030406

**Published:** 2020-03-01

**Authors:** Youssef A. Attia, Mohammed A. Al-Harthi, Mohamed A. Korish, Mohamed H. Shiboob

**Affiliations:** 1 Arid Land Agriculture Department, Faculty of Meteorology, Environment, and Arid Land Agriculture, King Abdulaziz University, Jeddah 21589, Saudi Arabia; malharthi@kau.edu.sa (M.A.A.-H.); mmkorish@yahoo.com (M.A.K.); 2 Environmental Department, Faculty of Meteorology, Environment and Arid land Agriculture, King Abdulaziz University, P.O. Box 80208, Jeddah 21589, Saudi Arabia; mshiboob@kau.edu.sa

**Keywords:** egg sources, amino acids ratios, antioxidant, recommended daily allowance

## Abstract

**Simple Summary:**

At present, great attention has been paid to the nutritional values of animal products to resilient humans against pathogens, boost their immunity, and cure diseases. Thus, this research investigated the nutritional value of four sources of commercial table eggs in the retail market in Jeddah, KSA, with the possible presence of raw protein, amino acid content, and protein quality indicators for different parts of eggs. The examined eggs showed a different percentage of essential and non-essential amino acids and antioxidant amino acids, suggesting a potential for enriching the nutritional values and prolonging the shelf life of the eggs by various nutritional strategic ways to enhance the antioxidant amino acids and the essential amino acid profile in eggs.

**Abstract:**

Considering the common believe that all eggs in the retail market are nutritionally similar, four different commercial sources of eggs (A, B, C, and D) available in a retail market were collected to investigate the crude protein and amino acid content, as well as the protein quality in the whole edible part of eggs (albumen + yolk), egg albumen, and egg yolk, separately. Five egg samples per source were collected four times during the experimental period, which resulted in a total number of 20 samples that were pooled to finally present five samples per source of eggs. The results show that crude protein in albumen was significantly higher in A and B than that of C and D, but the difference was found among edible parts of eggs such as yolk > whole edible part > albumen. Essential amino acids (arginine, histidine, isoleucine, lysine, methionine, methionine + cysteine, phenylalanine, phenylalanine + tyrosine, threonine, and valine) of eggs significantly differed according to the source of eggs, but eggs from different sources could provide from 17.4–26.7% of recommended daily allowance (RDA) of amino acids for adults. Essential amino acids (EAAs) were higher (*p* ≤ 0.05) in eggs from sources A and B than in source D, while source C exhibited intermediate values. Source B had greater (*p* ≤ 0.05) non-essential amino acids (NEAAs) than did sources C and D in whole edible egg, while source A displayed intermediate values. The phenylalanine + tyrosine, histidine, and lysine were the 1st, 2nd, and 3rd limiting amino acids in all sources of eggs. In conclusion, the investigated eggs showed different EAAs/NEAAs ratio and antioxidant amino acids, indicating a potential for enhancing nutritional values and extending the shelf life of eggs by different nutritional additions.

## 1. Introduction

Eggs are a portion of essential food for human consumption and contain most of the necessary nutrients, based on the daily need [1,2]. Eggs have all the crucial nutrients for life and are a valuable source of protein\amino acids, antioxidants, and bioactive components [3,4]. Furthermore, there has been considerable progress in the egg production industry, including the improvement of the genetic makeup of layers and production capacity, egg quality, and layers management [5,6]. 

Protein and amino acids are primary components of eggs and play critical roles in egg consumption and nutrition as they present the main part of the muscle, body function, hormones, enzymes and body fluids [7,8]. The total amino acids (TAAs) in eggs were 10.0 and 10.1 mg/g in the dry yolk of eggs in corn- and wheat-based diets, respectively [4]. According to the same authors, lysine was found to be 929, 1182 and 760 mg/100 g in fresh whole eggs, yolk, and albumen, respectively; for methionine, the corresponding values were 400, 375, and 396 [9]. The essential amino acids (EAAs) on dry yolk accounted for about 46–47%, while aromatic amino acids represented about 11% of the TAAs; arginine was the most copious amino acid (about 1.5 mg/g) in all samples, followed by glutamic acid (1.2 mg/g) and lysine (0.9 mg/g). The authors added that yolk samples content of the TAAs decreased by different cooking procedures, except for the boiled yolk of hens that were fed a corn-based diet, and tyrosine and tryptophan were noticed to be the key providers to the antioxidant properties of eggs. 

Eggs were also found to be a rich source of antioxidants [4,9] with the amino acid cysteine having the potential as an antioxidant amino acid, and the combination of strong, weak, and non-antioxidant amino acids increase the antioxidant capacity [9,10]. Besides, the amino acid content of eggs was found to be affected by poultry breeds [5] and species [6], preparation method [11,12] and components (whole, albumen, and yolk) of an egg [13]. The literature review indicates that there is room to improve the nutritional values of eggs by breeds and dietary and management practices due to the differences found in amino acids and antioxidant profiles of table eggs [1,5,6]. However, data for amino acids profile and amino acids ratio of eggs in the retail market are scarce, and there is a common belief that all eggs are nutritionally equal. The present research aims to investigate the crude protein and amino acid content and to evaluate the protein and amino acid ratio in commercial table eggs from four sources in the retail market in terms of recommended daily allowance (RDA) for adults.

## 2. Materials and Methods 

### 2.1. Sample Collection 

The Deanship of Scientific Research, King Abdulaziz University Saudi Arabia, approved the experimental procedures under protocol no. D-156-155-1438. It recommends animal rights, welfare, and minimal stress and did not cause any harm or suffering to animals, according to the Royal Decree M59 on 14/9/1431H.

Four commercial table egg sources, named A, B, C, and D in the retail market from white eggshell hybrids laying hens, originated from Single Comb White Leghorn commercially available in Saudi Arabia. The hens had 40–60 weeks of age. The hens were housed in cages in environmental control houses, and fed commercial layer diets contained 17% crude protein (CP), 11.60 MJ/kg, 3.5% Ca, and 0.35% none-phytate phosphorus kcal, in mash form and offered free access to feed and water and eliminated with 14:10 light-dark cycle. The medical care, vaccination, and husbandry practice were as suggested by the primary breeders and carried under the supervision of a veterinarian. 

Fresh eggs of grade A classes, stored at 5 °C, were gathered randomly to represent various sources of eggs in retail markets in Jeddah city, Saudi Arabia. The eggs were clean, of normal eggshell index, and of medium size, 55–60 g, of grade A. Thirty eggs were collected from A, B, C, D sources four times during February, March, April, and May 2017. The eggs of each source per time were broken opened, and five samples of each albumen, yolk, and albumen + yolk were randomly collected. Thus, there were 20 egg samples per time per egg part. After the last egg collection in May 2017, at 60 weeks of age, the samples (20 samples) of each egg source per part were pooled over time to finally present five samples per source of eggs per egg part albumen, yolk, and albumen + yolk, and thus used for protein and amino acids analyses.

### 2.2. Measurements

#### 2.2.1. Determination of Crude Protein

The protein content of eggs (yolk, albumen and yolk with albumen) was determined following method number 954.01, the principle of the Kjeldahl method [14]. One gram of sample was digested using 15 mL sulphuric acid (96%), utilizing an electrically heated block digester. The resultant digest was made alkaline by dilution with 50 mL of 40% sodium hydroxide. Thereafter the diluted sample was rapidly steam distilled for ammonia into 25 mL 4% boric acid. The sample was then manually titrated with 0.2 N hydrochloric acid. The protein content was estimated using 6.25 as a conversion factor of nitrogen to crude protein. Triplicate samples were analyzed, and the results are expressed as g/100 g dry matter basis of a sample [14]. 

#### 2.2.2. Determination of Amino Acids

The egg samples were dried using method number 934.01, and defatted using method number 920.39, as per reference [14]. A part of each sample, 0.2 g was hydrolyzed with 6 N HCl (10 mL) in a sealed tube and then heated in an oven at 100 °C for 24 h [15]. For the analysis of methionine and cysteine, samples were oxidized by performic acid before hydrolysis [16]. The resulting solution was brought to 25 mL with de-ionized water. After filtration, 5 mL of hydrolysate was evaporated until free from HCl. Then, the residue was dissolved in a diluting citrate buffer. The amino acids were measured using an Automatic Amino Acid Analyzer model AAA400 (Ingos Ltd., K Nouzovu 2090, 14316 Prague 4, Czech). The column was filled with Resin material and Ninhydrin reagent. The separation of amino acids depends on using different gradient pH buffers. Acid hydrolysis of samples was carried out following [15,17]. Tryptophan was determined, as reported by [18] and modified by [19]. 

#### 2.2.3. Predicted Protein Quality and Amino Acids Ratios 

The predicted protein efficiency ratio (P-PER) of the different sources of eggs was estimated from their amino acid content, according to [20]:

P-PER = −0.468 + 0.454 (Leucine) − 0.105 (Tyrosine)

#### 2.2.4. Amino Acids Ratios of Different Egg Sources

The quality of the amino acids was determined by estimating the ratio of amino acids in the egg samples compared with the requirements stated as a ratio [21]. The Amino Acid Score (AAS) was then predicted by applying the formula in reference [22].

The essential amino acid to TAAs ratio and the cystine to sulfur amino acids ratio were, thus calculated. The EAAs/non-essential amino acids (NEAAs) ratio was also calculated. The total of the aromatic amino acids was also estimated, and the ratio of aromatic amino acids to TAAs was calculated. 

Tyrosine + tryptophan to TAAs and total aromatic amino acids were also calculated as indices of the antioxidant property of eggs [4]. 

### 2.3. Statistical Analysis

The Data were evaluated using the one-way ANOVA of SAS software program [23] according to the following model:

Yij = µ + Ai + eij,

where: µ = general mean; Ai: influence of the source of the egg; eij: random error. The same model was used for the evaluation of amino acid patterns of different parts of the eggs. 

The percentage of data were normalized by applying transformations to arcsin before running the analysis. Means differences were compared using the Student-Newman Keuls test (*p* ≤ 0.05). 

## 3. Results and Discussion 

### 3.1. Growth Performance Crude Protein and Amino Acids Pattern of Whole Egg 

The content of the crude protein and amino acids in the whole edible part of the egg is presented in Table 1. Crude protein of whole eggs was not significantly different among different egg sources. The results are in agreement with those recently published by Secci et al. [24,25] Arginine, lysine, threonine, and proline were significantly greater in eggs from sources C and D than in those of A, B, but phenylalanine + tyrosine (aromatic amino acids along with histidine and tryptophan) showed the contrary trend. However, the difference between sources A and B in these amino acids was not significant. Tyrosine is conditional NEAAs and an important for photosynthesis and signal transduction processes [26]. Phenylketonuria is a genetic disease in which the tyrosine becomes EAAs because tyrosine cannot be synthesized from phenylalanine [26]. The EAAs/NEAAs were significantly greater for sources A, B, and then source D, and the latter was greater than source C. 

Glycine, am NEAA, was higher in the D source than in the C source. Isoleucine, methionine, and methionine + cystine were significantly higher in the B source than in other sources. In addition, source A showed greater isoleucine, methionine, and methionine + cysteine than did sources C and D, although source C exhibited higher isoleucine than did source D. 

Phenylalanine, an EAAs for the biosynthesis of norepinephrine and epinephrine, and valine, which is important amino acids for maintaining muscles, as well as for the regulation of the immune system were significantly higher in source B than in other sources. The other sources exhibited significant differences, showing a trend of B > A > D > C for phenylalanine and B > A > C > D for valine. Valine, along with leucine and isoleucine, are branched-chain amino acids and represented about two-thirds of amino acids in the body protein [27]. In whole edible parts of eggs, branched-chain amino acids amounted to ~45% of EAAs. Alanine, the 4th principal NEAAs, was similar in sources A and B, which was significantly greater than in sources C and D. The latter sources showed significant differences between them, with source C having higher. A similar trend was found for aspartic acid, but the differences between source C and other sources were not significant. 

Glutamic acid, the chief NEAA, was significantly greater in sources A, B, and C than in source D. Serine, which is important in biosynthesis of purine and pyrimidines, was significantly higher in source C than in the other sources, which showed significant differences among them in the following order: D > A > B. These findings reflected to some extent difference in amino acids in egg albumen and egg yolk and indicated that eggs from various sources have different amino acid patterns, which could positively affect the nutritional values and consumer preference [4,27]. 

### 3.2. Crude Protein and Amino Acid Patterns of Albumen 

Table 2 reports the crude protein and amino acid composition of the egg albumen. It was observed that the differences among the egg sources were significant for crude protein with sources A and B had usually higher values than sources C and D. Egg source shows a significant effect on some amino acids except for arginine, histidine (precursor for histamine and carnosine synthesis), lysine, threonine, tryptophan (important EAAs along with other EAAs in biosynthesis of protein), valine, alanine, aspartic, serine, and EAAs/NEAAs.

Leucine and phenylalanine were similar in sources A and B and markedly (*p* ≤ 0.05) higher than in source D, while the C source did not significantly differ from the other sources. Methionine and glutamic acid were significantly greater of sources A, B, and C than that of source D. Methionine + cystine, phenylalanine + tyrosine, EAAs, and proline were similar in sources A and B and significantly lower in sources C and D. Source D exhibited significantly lower values than source C. Glycine was significantly higher in source D than in the other sources. Non-essential amino acids were similar in sources A and B and significantly lower in sources C and D, with no significant differences between the latter sources. 

### 3.3. Crude Protein and Amino Acid Patterns of Egg Yolk

Table 3 shows the findings related to crude protein and amino acid profile of egg yolk. The difference in crude protein was not significant among various sources. The observed differences between the sources of eggs were significant (*p* ≤ 0.05) for some amino acids except for arginine, lysine, phenylalanine + tyrosine, threonine, tryptophan, valine, serine, and EAAs/NEAAs ratio. 

Isoleucine, leucine, aspartic acid, and glutamic acid were similar in sources A, B, and C, but significantly lower in source D. Methionine, phenylalanine, and proline were similar in eggs from sources A and B and in sources C and D, although differences between the former and the latter groups were remarkable (*p* ≤ 0.05) in favor of the former group. 

Methionine + cystine was significantly higher in source B than in sources A and C, while the C source had higher values than source A. Source D also had higher values than that of source A but did not significantly differ from sources B and C. Essential amino acids and alanine were significantly lower in source D than in sources A and B, while source C had intermediate values. In contrast, glycine was significantly higher in source D than in other sources. In addition, sources A and B showed similar values for glycine, which were greater than in source C. Glycine is a precursor to proteins and for the biosynthesis of collagen and considered the simplest amino acid that can be formed from serine and this reaction is reversible [28]. 

Essential amino acids were higher (*p* ≤ 0.05) in egg yolk from sources A and B than in source D, while source C exhibited intermediate values. Source B had greater (*p* ≤ 0.05) NEAAs than did sources C and D, while source A had intermediate values.

In general, the results indicated that EAAs were the highest in source A and B of both egg albumen and egg yolk. However, the EAAs/NEAAs ratio was not different among various sources of eggs. Leucine, followed by lysine and arginine, were the most abundant EAAs. Glutamic acid and aspartic acids were the predominant NEAAs in the whole edible parts of eggs and the egg yolk. The principal amino acids in egg albumen were leucine, lysine, and valine, with the glutamic acid and aspartic acid were the chief NEAAs. These results are similar to the results reported by [9], the sum of phenylalanine and tyrosine was found to have the highest values, whereas tryptophan displayed the lowest values in different parts of eggs. According to [3,5,29], animal protein is better digested and contains more EAAs and greater available AAs than vegetable protein. Besides, the protein quality of animal protein was higher than that of leguminous seeds [30,31]. 

### 3.4. Crude Protein and Amino Acid Patterns of Different Eggs Parts

Table 4. displays the crude protein and amino acid profile of different egg parts compared to the recommended daily allowances. Results showed variations (*p* ≤ 0.05) among different parts, e.g., the whole edible parts of eggs (albumen + yolk), albumen, and yolk. The yolk had a greater (*p* ≤ 0.05) concentration of crude protein and amino acids than the whole edible parts of eggs, which in turn had greater values (*p* ≤ 0.05) than the albumen. However, for the EAAs/NEAAs, the trend was opposite, being highest in the albumen and lowest in the yolk. The values for the whole edible parts of the eggs were intermediate.

Eggs are well-known animal proteins with high protein/amino acid quality that fulfil the human RDA better than vegetable protein sources, and EAAs deficiency can depress growth and lead to many health problems [2,26]. In this regard, different egg sources showed different patterns in phenylalanine, methionine, lysine, isoleucine, valine, and threonine in the whole edible parts of eggs. Differences in these amino acids were 38.5, 52.5, 16.4, 17.5, 20.9, and 21.1%, respectively. Similar trends were found in the albumen and yolk of eggs. These results agree with those reported by [6,11], who reported that raw regular Kampung eggs (eggs that produced by village hens that allowed to roam in a building, room or open area that includes nest space and perches, but they did have access to the outdoors) and nutrient-enriched eggs have different amino acid patterns, with lysine, leucine, phenylalanine, and valine having the highest concentrations in eggs. The difference in protein and AA patterns of various eggs could be attributed to the impact of the diet composition on the level of CP and amino acids. Similar results were reported by [1,5,6,7]. 

Results indicate that the yolk fulfils the highest percentage of the RDA for adults (70 kg) between 19–30 years of age, followed by the whole egg and albumen. For the egg yolk, the highest percentages were for threonine (81.5%), phenylalanine + tyrosine (78.8%), and tryptophan (78.3%) while the lowest was for methionine (~51.4%). The corresponding values for the whole edible parts of eggs and albumen were 58.5% and 41.9% for threonine, respectively, 66.8 and 53.3% phenylalanine + tyrosine, respectively, and 51.1% and 42.9% for tryptophan, respectively. 

From the results obtained for amino acid scores, phenylalanine + tyrosine was the most limiting, the amino acids that were deficient in protein and when supplemented resulted in the highest response, the amino acid in all sources of eggs, followed by histidine and lysine [32]. Limiting EAAs for humans that can’t be synthesized at an adequate amount by human cells are phenylalanine, methionine, lysine, leucine, isoleucine, valine, threonine, and tryptophan [11,33]. In addition, histidine, arginine, cystine and tyrosine are regarded as EAAs for infants and growing children [34]. 

From a biochemical point of view, differences in amino acid patterns in egg proteins/amino acids could be attributed to the type and percentage of protein in the albumen and yolk, e.g., ovalbumin, ovotransferrin, lysozyme, ovomucoid, ovomucin, and immunoglobulin Y [2,35]. The ovalbumin is the major protein that amounts to greater than 50% of the protein in the albumen of eggs [36]. In this regard, lysine (509 mg/g) was the greatest amino acid in eggs, whereas cysteine (128 mg/g) was the lowest [37]. Arginine, serine, cysteine and iso-leucine amino acids in eggs were higher than those in soy protein, beef, casein, wheat flour, and egg white [38].

### 3.5. Amino Acids Ratios

Table 5 shows the data for amino acid scores, aromatic amino acids, and essential amino acids to TAAs, antioxidant amino acid protein of eggs from different sources. The results indicate that the amino acid scores for histidine and threonine were significantly different among egg sources, with the highest values from source D and the lowest from source B. Differences in amino acid scores were also significant for the amino acids valine, isoleucine, leucine lysine, methionine + cystine, and phenylalanine + tyrosine. 

The present results show that source B had greater (*p* ≤ 0.05) amino acid scores for valine, isoleucine, and methionine + cystine than did sources C and D. In addition, source C had greater scores (*p* ≤ 0.05) for leucine, lysine, phenylalanine + tyrosine than did sources B and D, A and B and B, respectively. The results indicate that source C had the lowest quality of protein in terms of total aromatic amino acids (TAAAs) indices, but when tyrosine + tryptophan was considered as the main contributor to the antioxidant properties of eggs [4], source C had the highest value. Differences between the highest and lowest values for TAAAs and tyrosine + tryptophan were 7.4 and 8.5%, respectively. 

The EAAs/TAAs and cystine/sulfur amino acids were also higher in source C and D compared to the resources A and B; the differences in these criteria reached 4.7 and 45%, respectively, showing higher variability in cystine/sulfur amino acids as indices. The results also show greater amino acid scores in sources A and B, particularly for valine, isoleucine, and methionine + cystine. In contrast, the P-PER based on the content of leucine and tyrosine showed insignificant differences among different egg sources, indicating that this measurement is not a suitable parameter for protein quality evaluation of eggs, as the amino acids involved in the equation of calculation did not reflect the abundant amino acids= in eggs, suggesting a need for precise equation. 

The present results are in line with those cited by Adeyeye [6], who observed a TAAs of 10.0 and 10.1 mg/g of dry yolk in corn- and wheat-based diets, respectively. Moreover, differences in glutamic acid, glutamine, glycine, arginine, isoleucine, and ornithine were significant among dietary compositions. Similar to the present findings, EAAs accounted for about 46–47%, while TAAAs accounted for about 11% of the TAAs; arginine was the most copious amino acid, followed by glutamic acid and lysine [1,4]. Differences in the ratios of the amino acids of eggs from various sources could be due to the strains/breeds of layers and poultry species [5,6,39], dietary composition, cooking method, storage time, environment, [11,40,41] and egg parts [12,42,43].

The total aromatic amino acids, (TAAAs)/TAAs, as the index of antioxidant property of eggs [6], and EAAs/TAAs as an index of protein quality [42,43] were significantly different among various sources of eggs, with sources A and B having greater (*p* ≤ 0.05) values than source C. In addition, TAAAs/TAAs were significantly greater in source D than in source C. Tyrosine + tryptophan, as an absolute value or relative to TAAAs or TAAs, was significantly different among different egg sources. The C sources had greater values (*p* ≤ 0.05) than B sources. In addition, sources A and B showed similar values as source C except for tyrosine + tryptophan/TAAAs, which was higher (*p* ≤ 0.05) in source C. No significant (*p* ≥ 0.05) variations in the predicated protein efficiency ratio (P-PER) ratio among various egg sources. The cystine/sulfur amino acids ratio was significantly higher for eggs of source C.

## 4. Conclusions

In conclusion, the investigated eggs showed different EAAs/NEAAs ratio and antioxidant amino acids, indicating a potential for enhancing the nutritional values and extending the shelf life of eggs by different strategic nutritional approaches such as increasing antioxidants, and essential amino acid content of eggs.

## Figures and Tables

**Table 1:** Crude protein and amino acid patterns of whole edible parts of eggs of different sources in the retail market.

Crude Protein (g/100 g)/Amino Acid (mg/100 g)	Source of Eggs				Statistical Analysis	
	A	B	C	D	RMSE	*p*-Value
Crude protein	14.03	14.02	13.77	13.88	0.395	0.721
Arginine	769.0 ^b^	693.3 ^b^	915.3 ^a^	882.3 ^a^	61.65	0.001
Histidine	296.8 ^bc^	279.0 ^c^	315.8 ^ab^	331.8 ^a^	12.94	0.001
Isoleucine	698.3 ^b^	720.0 ^a^	641.8 ^c^	613.3 ^d^	10.82	0.001
Leucine	1100.0	1081.3	1128.0	1057.8	36.19	0.097
Lysine	923.0 ^b^	870.3 ^c^	1019.3 ^a^	1012.8 ^a^	19.21	0.001
Methionine	394.3 ^b^	434.3 ^a^	301.8 ^c^	285.0 ^c^	16.87	0.001
Methionine + cysteine	554.5 ^b^	627.5 ^a^	513.5 ^c^	490.0 ^c^	30.01	0.002
Phenylalanine	679.8 ^b^	744.5 ^a^	537.8 ^d^	618.3 ^c^	32.51	0.001
Phenylalanine + tyrosine	1199.0 ^a^	1237.8 ^a^	1099.0 ^b^	1137.8 ^b^	30.01	0.002
Threonine	613.0 ^b^	578.3 ^b^	674.8 ^a^	699.5 ^a^	23.48	0.001
Tryptophan	147.0	145.5	132.0	148.0	9.04	0.094
Valine	781.8 ^b^	815.3 ^a^	707.8 ^c^	673.8 ^d^	15.91	0.001
EAAs	5634.0 ^b^	5668.3 ^a^	5458.0 ^c^	5439.5 ^c^	106.4	0.021
Alanine	713.0 ^a^	737.3 ^a^	651.0 ^b^	558.0 ^c^	20.89	0.001
Aspartic acid	1295.8 ^a^	1310.5 ^a^	1260.0 ^ab^	1218.5 ^b^	36.66	0.019
Glutamic acid	1687.0 ^a^	1713.5 ^a^	1642.8^a^	1555.3 ^b^	37.32	0.001
Glycine	426.0 ^ab^	441.3 ^ab^	381.8 ^b^	473.8 ^a^	32.42	0.014
Proline	507.0 ^b^	493.3 ^b^	523.8 ^a^	535.8 ^a^	10.75	0.001
Serine	956.3 ^c^	881.5 ^d^	1099.8 ^a^	1055.3 ^b^	23.54	0.001
NEAAs	7033.5	6957.5	7247.0	7001.8	167.9	0.129
EAAs/NEAAs	0.801 ^a^	0.815 ^a^	0.753 ^c^	0.777 ^b^	0.0214	0.011

^a–d^ means with varying superscripts differ significantly (*p* < 0.05); RMSE, Root mean square error; *p* value, probability level; EAAs, total essential amino acids; NEAAs, total non-essential amino acids; EAAs/NEAAs, total essential amino acids/total non-essential amino acids ratio.

**Table 2:** Crude protein and amino acid patterns of albumen of eggs of different sources in the retail market.

Crude Protein (g/100 g)/Amino Acid (mg/100 g)	Source of Eggs				Statistical Analysis	
	A	B	C	D	RMSE	*p*-Value
Crude protein	12.33 ^a^	12.45 ^a^	11.73 ^b^	11.83 ^b^	0.536	0.036
Arginine	543.5	544.5	516.5	497.8	38.16	0.293
Histidine	221.8	222.8	194.8	198.8	19.16	0.130
Isoleucine	566.5	567.5	567.0	503.3	31.44	0.035
Leucine	845.0 ^a^	845.8 ^a^	818.3 ^ab^	767.8 ^b^	35.79	0.033
Lysine	745.8	746.8	743.5	738.0	25.48	0.962
Methionine	342.5 ^a^	343.5 ^a^	315.3 ^a^	262.5 ^b^	17.28	0.001
Methionine + Cystine	497.3 ^a^	499.3 ^a^	443.5 ^b^	381.5 ^c^	25.30	0.001
Phenylalanine	583.8 ^a^	584.8 ^a^	556.8 ^ab^	519.5 ^b^	25.24	0.012
Phenyalanine + Tyrosine	972.6 ^a^	974.3 ^a^	918.0 ^b^	868.0 ^c^	30.22	0.001
Threonine	454.5	455.5	427.3	424.0	27.11	0.251
Tryptophan	118.0	118.8	117.8	132.0	15.53	0.354
Valine	613.0	588.5	585.8	564.8	62.73	0.758
EAAs	4489.8 ^a^	4473.5 ^a^	4320.8 ^b^	4110.8 ^c^	76.45	0.001
Alanine	577.8	578.5	552.0	581.3	47.12	0.799
Aspartic acid	1023.5	1024.5	996.3	1035.3	29.22	0.325
Glutamic acid	1336.5 ^a^	1337.8 ^a^	1309.8 ^a^	1066.5 ^b^	34.65	0.001
Glycine	347.8 ^b^	348.8 ^b^	321.0 ^b^	598.0 ^a^	22.65	0.001
Proline	388.5 ^a^	389.5 ^a^	361.3 ^b^	328.8 ^c^	14.57	0.003
Serine	689.8	690.8	663.0	662.3	18.41	0.077
NEAAs	5450.8 ^a^	5459.7 ^a^	5209.8 ^b^	5137.5 ^b^	130.8	0.009
EAAs/NEAAs	0.824	0.819	0.829	0.800	0.0261	0.423

^a–c^ means with varying superscripts differ significantly (*p* < 0.05); RMSE, Root mean square error; *p* value, probability level; EAAs, total essential amino acids; NEAAs, total non-essential amino acids; EAAs/NEAAs, total essential amino acids/total non-essential amino acids ratio.

**Table 3:** Crude protein and amino acid patterns of the yolk of eggs of different sources in the retail market.

Crude Protein (g/100 g)/Amino Acid (mg/100 g)	Source of Eggs				Statistical Analysis	
	A	B	C	D	RMSE	*p*-Value
Crude protein	15.97	16.12	15.83	16.04	0.796	0.873
Arginine	1156.3	1150.0	1133.8	1131.3	24.78	0.440
Histidine	418.3 ^a^	412.0 ^a^	393.3 ^ab^	375.5 ^b^	16.68	0.016
Isoleucine	819.8 ^a^	813.0 ^a^	795.3 ^a^	712.5 ^b^	19.11	0.001
Leucine	1418.0 ^a^	1411.8 ^a^	1393.3 ^a^	1317.5 ^b^	15.49	0.001
Lysine	1284.3	1278.0	1236.8	1243.5	33.96	0.174
Methionine	401.0 ^a^	394.8 ^a^	353.5 ^b^	348.8 ^b^	19.27	0.004
Methionine + cysteine	531.8 ^c^	678.8 ^a^	598.8 ^b^	624.5 ^ab^	36.01	0.001
Phenylalanine	691.8 ^a^	685.0 ^a^	655.8 ^b^	644.3 ^b^	17.53	0.008
Phenylalanine + tyrosine	1412.5	1399.0	1363.0	1342.0	36.99	0.073
Threonine	860.3	854.0	822.3	888.0	31.62	0.082
Tryptophan	185.5	218.5	263.8	208.8	35.89	0.059
Valine	860.8	854.3	865.8	878.0	58.07	0.947
EAAs	6939.8 ^a^	6921.5 ^a^	6768.0 ^ab^	6628.5 ^b^	112.7	0.008
Alanine	831.0 ^a^	824.8 ^a^	788.5 ^ab^	761.3 ^b^	22.40	0.003
Aspartic acid	1580.8 ^a^	1574.3 ^a^	1547.8 ^a^	1492.3 ^b^	33.99	0.014
Glutamic acid	2051.8 ^a^	2045.0 ^a^	2024.3 ^a^	1958.0 ^b^	27.41	0.002
Glycine	499.8 ^b^	493.3 ^b^	464.8 ^c^	537.8 ^a^	14.36	0.002
Proline	674.3 ^a^	668.0 ^a^	632.5 ^b^	626.8 ^b^	20.74	0.015
Serine	1343.5	1377.0	1335.8	1315.3	38.71	0.212
NEAAs	8988.5 ^ab^	9131.0 ^a^	8864.5 ^b^	8811.5 ^b^	115.1	0.011
EAAs/NEAAs	0.772	0.758	0.763	0.752	0.0169	0.481

^a–c^ Means with varying superscripts differ significantly (*p* < 0.05); RMSE, Root mean square error; *p* value, probability level; EAAs, total essential amino acids; NEAAs, total non-essential amino acids; EAAs/NEAAs, total essential amino acids/total non-essential amino acids ratio.

**Table 4:** Amino acid patterns of different egg parts in the retail market compared to recommended daily allowance standard Food and Agriculture Organization (FAO)/World Health Organization (WHO) [22] values of adults (70 kg).

Crude Protein (g/100 g)/Amino Acid (mg/100 g)	Egg Part			Statistical Analysis		RDA, mg/kg Day
	Whole Eggs	Albumen	Yolk	RMSE	*p*-Value	
Crude protein	13.95 ^b^ (26.6%)	12.42 ^c^ (23.7%)	16.00 ^a^ (30.5%)	3.32	0.054	0.75 g/kg per day
Arginine	814.9 ^b^	525.6 ^c^	1142.8 ^a^	43.93	0.001	--
Histidine	312.3^b^ (44.6%)	209.5^c^ (29.9%)	399.8 ^a^ (57.1%)	16.33	0.001	10 (700 mg/day)
Isoleucine	668.3 ^b^ (47.7%)	551.1 ^c^ (39.4%)	785.1 ^a^ (56.1%)	22.63	0.001	20 (1400 mg/day)
Leucine	1091.8 ^b^ (40.0%)	819.2 ^c^ (30.0%)	1385.1 ^a^ (70.7%)	31.42	0.001	39 (2730 mg/day)
Lysine	956.3 ^b^ (45.5%)	743.5 ^c^ (35.4%)	1260.6 ^a^ (60.0%)	27.13	0.001	30 (2100 mg/day)
Methionine	353.8 ^b^ (48.6%)	315.9 ^c^ (43.4)	374.5 ^a^ (51.4)	18.94	0.001	10.4 (728 mg/day)
Methionine + cysteine	546.4 ^b^ (52.0%)	455.4 ^c^ (43.4%)	608.4 ^a^ (57.9%)	29.99	0.001	15 (1050 mg/day)
Phenylalanine	645.1 ^b^	516.2 ^c^	669.2 ^a^+	27.64	0.001	--
Phenyalanine + tyrosine	1168.4 ^b^ (66.8%)	933.1 ^c^ (53.3%)	1379.1 ^a^ (78.8%)	34.73	0.001	25 (1750 mg/day)
Threonine	614.4 ^b^ (58.5%)	440.3 ^c^ (41.9%)	856.1 ^a^ (81.5%)	27.87	0.001	15 (1050 mg/day)
Tryptophan	143.1 ^b^ (51.1%)	120.1 ^c^ (42.9%)	219.1 ^a^ (78.3%)	23.13	0.001	4 (280 mg/day)
Valine	744.6 ^b^ (40.9%)	588.0 ^c^ (32.3%)	864.7 ^a^ (47.5%)	49.06	0.001	26 (1820 mg/day)
EAAs	5549.9 ^b^	4348.7 ^c^	6814.4 ^a^	104.2	0.001	--
Alanine	664.8 ^b^	572.4 ^c^	801.4 ^a^	31.78	0.001	--
Aspartic acid	1271.2 ^b^	1019.9 ^c^	1548.8 ^a^	33.79	0.001	--
Glutamic acid	1649.6 ^b^	1262.6 ^c^	2019.8 ^a^	33.52	0.001	--
Glycine	430.7 ^b^	378.8 ^c^	498.9 ^a^	23.81	0.001	--
Proline	514.9 ^b^	367.1 ^c^	650.4 ^a^	15.51	0.001	--
Serine	998.2 ^b^	676.4 ^c^	1342.9 ^a^	28.39	0.001	--
NEAAS	7059.9 ^b^	5314.4 ^c^	8948.9 ^a^	136.2	0.001	--
EAAs/NEAAs	0.786 ^b^	0.818 ^a^	0.761 ^c^	0.022	0.001	--

^a–c^ Means with varying superscripts differ significantly (*p* < 0.05); RMSE, Root mean square error; *p* value, probability level; FAO/WHO, Food and Agriculture Organization/World Health Organization; RDA, recommended daily allowance; EAAs, total essential amino acids; NEAAs, total non-essential amino acids; EAAs/NEAAs, total essential amino acids/total non-essential amino acids ratio.

**Table 5:** Amino acids score (%), aromatic amino acids, essential amino acids to the total amino acid ratio. Antioxidant amino acids of the whole edible egg parts (albumen + yolk) of different sources compared to standard Food and Agriculture Organization (FAO)/World Health Organization (WHO) [22] values.

Amino Acid (mg/100 g)	Source of Eggs				FAO/WHO, 2007 (g/100 g Protein)	Statistical Analysis	
	A	B	C	D		RMSE	*p*-Value
Amino Acid Score							
Histidine	95.3 ^ab^	89.5 ^b^	101.4 ^ab^	106.5 ^a^	1.5	3.26	0.001
Valine	136.2 ^ab^	141.9 ^a^	123.3 ^b^	117.4 ^b^	3.9	4.16	0.025
Isoleucine	152.0 ^a^	156.8 ^a^	139.8 ^b^	133.5 ^b^	3.0	5.14	0.004
Leucine	101.6 ^a^	99.9 ^b^	104.2 ^a^	97.7 ^b^	5.9	2.93	0.005
Lysine	97.0 ^b^	91.5 ^b^	107.2 ^a^	106.5 ^a^	4.5	3.87	0.002
Methionine + cysteine	292.4 ^ab^	301.9 ^a^	268.3 ^b^	277.6 ^b^	2.2	5.29	0.001
Phenylalanine + tyrosine	50.2 ^ab^	47.7 ^b^	54.3 ^a^	50.3 ^ab^	3.8	1.56	0.001
Threonine	109.9 ^b^	105.5 ^b^	121.1 ^a^	125.5 ^a^	2.3	4.56	0.003
Protein quality indices							
TAAAs	1643 ^a^	1662 ^a^	1547 ^b^	1618 ^ab^	---	46.5	0.024
TAAAs/TAAs ratio	0.1297 ^a^	0.1317 ^a^	0.1217 ^b^	0.1300 ^a^	---	0.003	0.001
Tyrosine + tryptophan	666.3 ^ab^	638.8 ^b^	693.3 ^a^	667.5 ^ab^	---	22.66	0.042
Tyrosine + tryptophan/TAAs ratio	0.0526 ^a^	0.0506 ^b^	0.0546 ^a^	0.0536 ^a^	---	0.001	0.001
Tyrosine + tryptophan/TAAAs ratio	0.406 ^b^	0.384c	0.448 ^a^	0.413 ^b^	---	0.067	0.003
EAAs/TAAs ratio	0.445 ^a^	0.449 ^a^	0.429 ^b^	0.437 ^ab^	---	0.007	0.009
P-PER	3.15	3.10	3.21	2.99	---	1.53	0.196
Cystine/sulfur amino acids ratio	0.289 ^b^	0.308 ^b^	0.412 ^a^	0.419 ^a^	---	0.015	0.001

^a–c^ means with varying superscripts differ significantly (*p* < 0.05); RMSE, Root mean square error; *p*-value, probability level; FAO/WHO, Food and Agriculture Organization/World Health Organization; EAAs, total essential amino acids; TAAAs, Total aromatic amino acids, TAAAs/TAAs, Total aromatic amino acids/total amino acids, EAAs/TAAs, essential amino acids/total amino acids, P-PER, predicated protein efficiency ratio.
